# Effects of Chloride Concentration on the Water Disinfection Performance of Silver Containing Nanocellulose-based Composites

**DOI:** 10.1038/s41598-019-56009-6

**Published:** 2019-12-20

**Authors:** Janika Lehtonen, Jukka Hassinen, Riina Honkanen, Avula Anil Kumar, Heli Viskari, Anu Kettunen, Nikolaos Pahimanolis, Thalappil Pradeep, Orlando J. Rojas, Olli Ikkala

**Affiliations:** 1 Department of Bioproducts and Biosystems, School of Chemical Engineering, Aalto University, P. O. Box 16300, FI-00076 Aalto Espoo, Finland; 2 Department of Applied Physics, School of Science, Aalto University, P. O. Box 16300, FI-00076 Aalto Espoo, Finland; 3 Industrial Water Ltd., Moreenikatu 2 B, FI-04600 Mäntsälä, Finland; 4 DST Unit of Nanoscience (DST UNS) and Thematic Unit of Excellence (TUE), Department of Chemistry, Indian Institute of Technology Madras, Chennai, 600036 India; 5 Betulium Ltd., Tekniikantie 2, FI-02150 Espoo, Finland

**Keywords:** Sustainability, Antimicrobials, Nanoscale materials, Organic-inorganic nanostructures, Synthesis and processing, Materials chemistry, Nanocomposites, Chemistry

## Abstract

The availability of microbially-safe drinking water is a challenge in many developing regions. Due to the well-known antibacterial effect of silver ions, materials used for their controlled release have been widely studied for point-of-use water disinfection. However, even if it is in principle known that chloride anions can suppress the antibacterial efficiency of silver, the majority of previous studies, surprisingly, have not focused on chloride concentrations relevant for freshwaters and thus for practical applications. Here, we prepared low-cost nanocellulose-aluminium oxyhydroxide nanocomposites functionalized with silver nanoparticles. Field samples obtained from Chennai, India were used as a guideline for choosing relevant chloride concentrations for the antibacterial studies, i.e., 10, 90, and 290 ppm. The antibacterial performance of the material against *Escherichia coli* and *Bacillus subtilis* was demonstrated and the influence of chloride concentration on the antibacterial effect was studied with *E. coli*. A 1 h contact time led to bacterial reductions of 5.6 log_10_, 2.9 log_10_, and 2.2 log_10_, respectively. This indicates that an increase of chloride concentration leads to a substantial reduction of antibacterial efficiency, even within chloride concentrations found in freshwaters. This work enables further insights for designing freshwater purification systems that utilize silver-releasing materials.

## Introduction

Waterborne diseases caused by micro-organisms are a major cause of death worldwide and a grand challenge remains to find sustainable solutions for their control and elimination^1^. The need for affordable, efficient, eco-friendly, and easily applicable technologies for removing micro-organisms is urgent. Point-of-use (POU) water treatment is often used when drinking water is retrieved from untreated natural sources^2^ where centralized water treatment facilities are not available^3^. Chlorine treatment is a generic method for household water disinfection, but the formation of toxic byproducts makes it problematic^4^, resulting in a need for alternative methods. Nanotechnology and especially nanoparticles have been studied as a possible solution to challenges related to water disinfection^5,6^. The use of silver as an antibacterial agent dates back already to the first century BC^7^. Nowadays, nanosilver is used in a variety of consumer products^5,8,9^ and these products are generally based on silver nanoparticles (AgNPs) that can be produced by chemical reduction of silver salts^10^. Several different materials containing silver in its colloidal form, particularly nanoparticles, have been studied for POU water treatment^3,11,12^. Chitosan cryogels^13^, cellulosic material such as paper filters^14^, alginate beads^3^, ceramic cubes^15^ and filters^16,17^, poly (sodium acrylate) cryogels^18^, and cotton textiles^6^ loaded with AgNPs have been demonstrated for POU water treatment. However, research in the field still involves subtleties of e.g. controlling the concentration of released silver ions and the economic feasibility of the materials.

A low-cost material based on chitosan and aluminium oxyhydroxide embedded with AgNPs has previously been developed for a gravity-driven filtration unit^19^. Inspired by that work, and to improve the economic feasibility of the product, we synthesized and characterized a composite material comprising of commercially available cationic cellulose nanofibrils (cCNF) and aluminium oxyhydroxide, which can retain AgNPs. Nanocellulose, including cellulose nanofibrils (CNF), is a renewable high strength nanoscopic material with interesting properties such as relatively high aspect ratio and broad range of easily applicable chemical modifications^20^, making it, e.g., an attractive reinforcing component for nanocomposites. CNFs are commonly prepared by mechanical treatments combined with chemical treatments and the resulting nanofibrils have lateral dimension in the order of 5–50 nm, depending on the source and preparation method. The chemical modification results in functionalization of the fibrils^21^, as for example in this work, with cationic quaternary ammonium groups. Importantly, cCNF was chosen as the starting material instead of unmodified CNF due to its inherent antibacterial properties^21,22^, thus further promoting the intended function of the composite material. The fabricated materials act as carriers for the AgNPs and release Ag^+^ when brought into contact with water.

The mechanism of action of AgNPs for causing antibacterial effect is not yet fully understood^10,23^. However, there is consensus that the oxidation of metallic silver to silver ions (Ag^+^) is required for achieving an antibacterial effect^7,24,25^, although nanoparticle-specific effects have also been considered as one cause of toxicity^10,26^. AgNPs have been shown to be non-toxic to *Escherichia coli* when conducting tests under strictly anaerobic conditions, where Ag^+^ release is precluded, thus suggesting that antibacterial activity of AgNPs would solely be due to Ag^+^ release^27^. Factors affecting the release kinetics of Ag^+^ from AgNPs include size of particles, temperature, shape^28^, the surface ligand^29^, and the composition of the surrounding medium^7^.

Silver ions can form complexes with different anions commonly present in freshwaters, thereby reducing the amount of free Ag^+^ in water. Chloride concentration ([Cl^−^]) has been recognized as a significant factor decreasing the antibacterial performance of silver ions due to the low solubility product of AgCl. In addition, complexation with e.g. sulfide and organic matter in freshwaters can result in decrease of free Ag^+^ concentration^30^. In freshwaters, [Cl^−^] can vary significantly. Still, this factor has not been properly considered when investigating the efficiency of silver-releasing materials for POU water purification. An objective of this study was first to screen realistic chloride concentrations relevant for applications and then to explore the effect of salinity in simulated freshwaters and more specifically, [Cl^−^], on the antibacterial effect of the composites on *Escherichia coli*, a bacterium typically used as a biological indicator of drinking water safety^31^. *Bacillus subtilis* was also used as a reference for a gram-positive model bacterium.

Unfortunately, the antibacterial tests of silver-releasing materials in laboratory conditions are often conducted in solutions with [Cl^−^] irrelevant to freshwaters in rural areas. The tests are often performed in very low [Cl^−^], such as highly diluted buffer solution or deionized water^4,32^, or very high [Cl^−^] such as undiluted PBS buffer where [Cl^−^] is typically around 5000 ppm^33^. When silver concentration is high, charged silver polychloride complexes are formed, which can contribute to the antibacterial activity^34^. Additionally, several studies where the influence of [Cl^−^] on AgNP toxicity has been considered, do not use [Cl^−^] occurring in freshwaters as a basis for choosing the studied conditions, resulting in studies with low concentrations such as 12.5 ppm and high concentrations such as around 5000 ppm^24,25,35^. Therefore, the fact that [Cl^−^] can vary significantly in freshwater sources and that this can affect silver-based water disinfection is the main issue we highlight in this work.

In this work, we used simulated freshwaters with [Cl^−^] in the range relevant to possible rural drinking water sources. Two different contact times (1 h and 2 h) between the composite and water were used. First, the antibacterial activity against *E. coli* and *B. subtilis* was compared and tests were continued with *E. coli* to study the influence of [Cl^−^] on the antibacterial activity. Silver release from the composite as a function of time was also studied. Finally, based on our observations, we give an evaluation of the potential of the composites for use in water disinfection.

## Results

### Characterization

**Figure 1:**
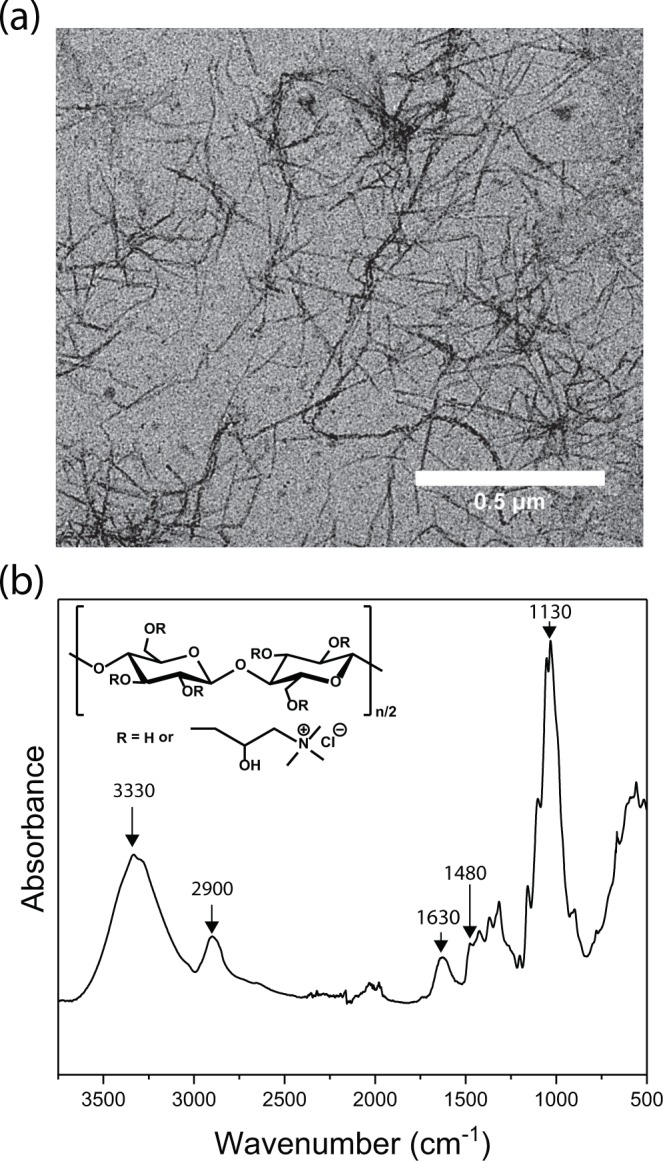
Characterization of cationic CNF (cCNF). (**a**) TEM image and (**b**) FTIR spectrum of cCNF (inset: chemical structure of cCNF).

The cCNF (see Methods) was provided by Betulium Ltd. and was first imaged by transmission electron microscopy (TEM) (Fig. 1a). The material was found to consist of fibrils with widths ranging from 5 to 20 nm and lengths up to few µm. Data from the quantification of ammonium groups on the fibrils by conductometric titration was provided by the manufacturer (0.63 mmol/g). ζ-potential measurements (Supplementary Fig. S1) confirmed the positive surface charge of cCNF in the whole pH range tested (pH 3–11). FTIR analysis of cCNF (Fig. 1b) revealed typical absorption peaks of cellulose (ν(O–H) at 3330 cm^−1^, ν_asym_(C–H) and ν_sym_(C–H) at 2900 cm^−1^, δ(H_2_O) at 1630 cm^−1^ and ν(C–O) at 1130 cm^−1^). The absorption at 1480 cm^−1^ was assigned to the methyl group of quaternary ammonium units. cCNF was chosen as the starting material due to its inherent antibacterial properties and economic feasibility. Composites were also prepared from unmodified CNF but preliminary tests indicated possible promotion of bacterial growth, thus suggesting to select cCNF.

**Figure 2:**
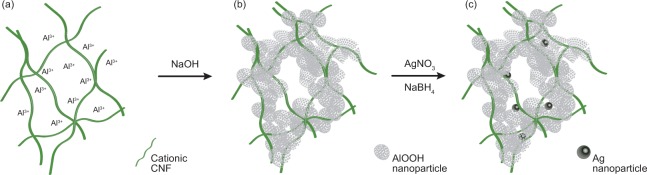
Synthesis of the cCNFAl and cCNFAl_Ag_ composites. (**a**) Solution of Al_2_(SO_4_)_3_ and cationic CNF, where Al^3+^ complexes with the fibrils. (**b**) Precipitation of the material with NaOH and the formation of AlOOH. (**c**) Synthesis of embedded AgNPs into the composite matrix.

cCNF together with Al_2_(SO_4_)_3_ was used to produce both composites without AgNPs (cCNFAl) and with AgNPs (cCNFAl_Ag_). Aluminium is present as hydrated Al^3+^ ions at low pH. An alkali treatment during the synthesis of the composite results in the formation of aluminium hydroxide, and aging the precipitate leads to the formation of aluminium oxyhydroxide (AlOOH)^36^. The synthesis process is described in Fig. 2. The phases in the cCNFAl_Ag_ composite were identified by X-ray diffraction (XRD) (Supplementary Fig. S2). The XRD pattern of the cCNFAl_Ag_ composite prepared from high purity Al_2_(SO_4_)_3_ hydrate shows reflections coinciding with the crystalline AlOOH boehmite phase^37^. The widths of the diffraction peaks suggest the size of the crystallites to be in the range of 4–5 nm based on the Scherrer equation. To ensure the economic feasibility of the end product, further experiments were focused on a composite prepared from technical grade Al_2_(SO_4_)_3_ hydrate. Therein, the precipitate was in an amorphous form^37^. However, both these materials could accommodate AgNPs and thus worked in the antibacterial test. The reflections from AgNPs were not resolved due to the small concentration of silver in the composite (0.67 wt-%). However, from a sample containing 1.12 wt-% silver, the reflections could be detected, and the size of silver crystallites could be estimated to be 15 nm based on the Scherrer equation.

**Figure 3:**
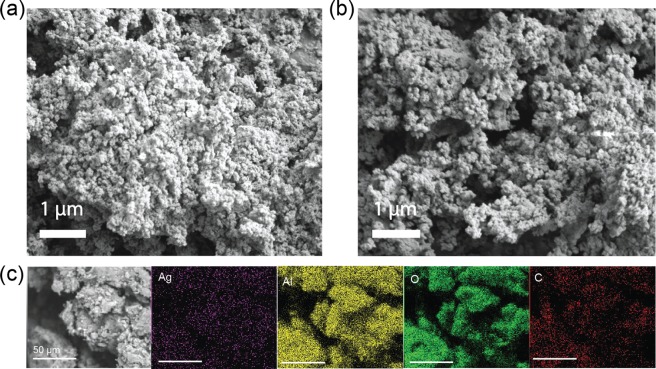
SEM images of composite materials. (**a**) cCNFAl nanocomposite, (**b**) cCNFAl_Ag_ nanocomposite and (**c**) SEM-EDX elemental mapping of cCNFAl_Ag_ nanocomposite (all scale bars are 50 µm).

Scanning electron microscope (SEM) images of both the composites (Fig. 3a,b) showed qualitatively similar porous structures indicating that the *in situ* AgNP synthesis did not cause any significant overall changes to the morphology of the material. More concrete proof of the similarities of the porous structure was provided by the high BET surface areas of the composites, which were 150 m^2^/g for both compositions. SEM with energy-dispersive X-ray spectroscopy (EDX) (Fig. 3c and Supplementary Fig. S3) was used for elemental analysis and the elemental mapping confirmed the presence of silver in the composite. The surface charge of the composite was determined by conductometric titration from the cCNFAl composite to avoid interference from the Ag^+^ dissolution from the AgNPs. The charge was 0.8 mmol/g indicating a positive surface charge of the composite (Supplementary Fig. S4).

### Antibacterial tests

The antibacterial effect of AgNPs against various Gram-negative and Gram-positive bacteria is well known^38–41^. However, the precise mechanism of the action of AgNPs on micro-organisms is not yet fully understood but protein inactivation and DNA damage have been suggested as possible causes^1,8,38^. Especially, interaction with thiol groups has been related to the antibacterial mechanism of silver^5,42^. Ag^+^ has also been shown to damage the cell membrane^1,5,43,44^. Both model organisms used in this work, Gram-negative *E. coli* and Gram-positive *B. subtilis*, are widely used in antibacterial studies. *E. coli* is a common cause of diarrheal diseases^45^ and can survive in various environments^46^ and is therefore a good reference bacterium to test materials for water purification. The compositions of the simulated freshwaters used for the antibacterial tests in this work are presented in Supplementary Table S1.

#### Antibacterial tests in simulated freshwater using *E. coli* and *B. subtilis*

**Figure 4:**
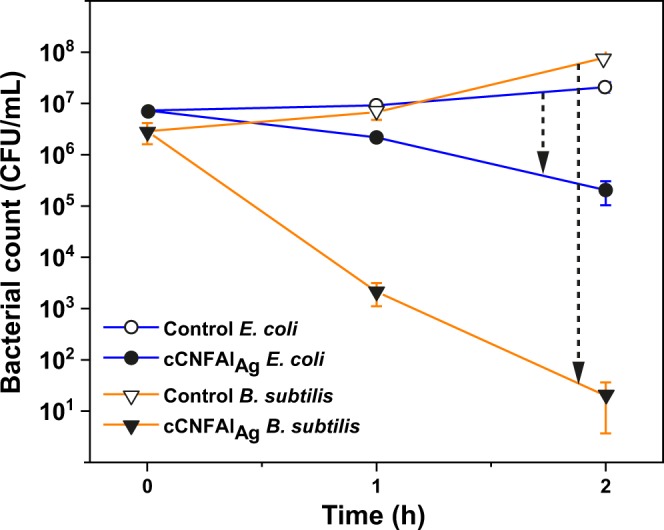
Antibacterial effect of the cCNFAl_Ag_ composite against *E. coli* and *B. subtilis* in simulated freshwater (Cl^−^_120ppm+nutrient_).

First, the antibacterial effectiveness of the cCNFAl_Ag_ composite on the two different bacteria was compared in simulated freshwater with 1% of Luria Bertani (LB) medium (Fig. 4). The small amount of medium introduced into the simulated freshwater with the inoculation of bacteria was found to be necessary to ensure the viability of *B. subtilis* for the duration of the experiment. This resulted in a final [Cl^−^] of 120 ppm, referred to as Cl^−^_120ppm+nutrient_. The data indicates that the antibacterial effect was more pronounced in the case of *B. subtilis* compared to *E. coli* (6.6 log_10_ reduction compared to 2 log_10_ reduction after 2 h, respectively). It can be also observed that the small amount of nutrient caused bacterial growth in the control tests without the composite. Interestingly, the growth of *B. subtilis* was faster than the growth of *E. coli* in the control tests. The lower initial concentration and faster growth rate of *B. subtilis* compared to *E. coli* could have enhanced the antibacterial effect. Stronger antibacterial activity of AgNPs or AgNP-embedded materials against *B. subtilis* compared to *E. coli* has also been observed in some previous studies^18,43,47^. However, also contradictory results, such as better activity of AgNP-containing methacrylic acid copolymer beads on Gram-negative bacteria compared to Gram-positive bacteria, have been reported^48^.

#### Effect of [Cl^−^] on antibacterial activity

In this study, the goal was to focus on [Cl^−^] relevant to freshwaters and typical drinking water sources. Therefore, the [Cl^−^] and *E. coli* concentrations were analyzed from samples collected from possible drinking water sources in the Indian Institute of Technology Madras campus, Chennai, India (Supplementary Table S2). These results indicate that there can be significant variations in [Cl^−^] of possible drinking water sources. Based on the collected data, 10 ppm, 90 ppm and 290 ppm, referred to as Cl^−^_10ppm_, Cl^−^_90ppm_, and Cl^−^_290ppm_, respectively, were selected to represent typical [Cl^−^] in purified drinking water, surface water, and well water, respectively. In addition, Cl^−^_290ppm_ coincides with the taste threshold of Cl^−^, therefore being the highest concentration relevant to drinking water purposes^49^.

**Figure 5:**
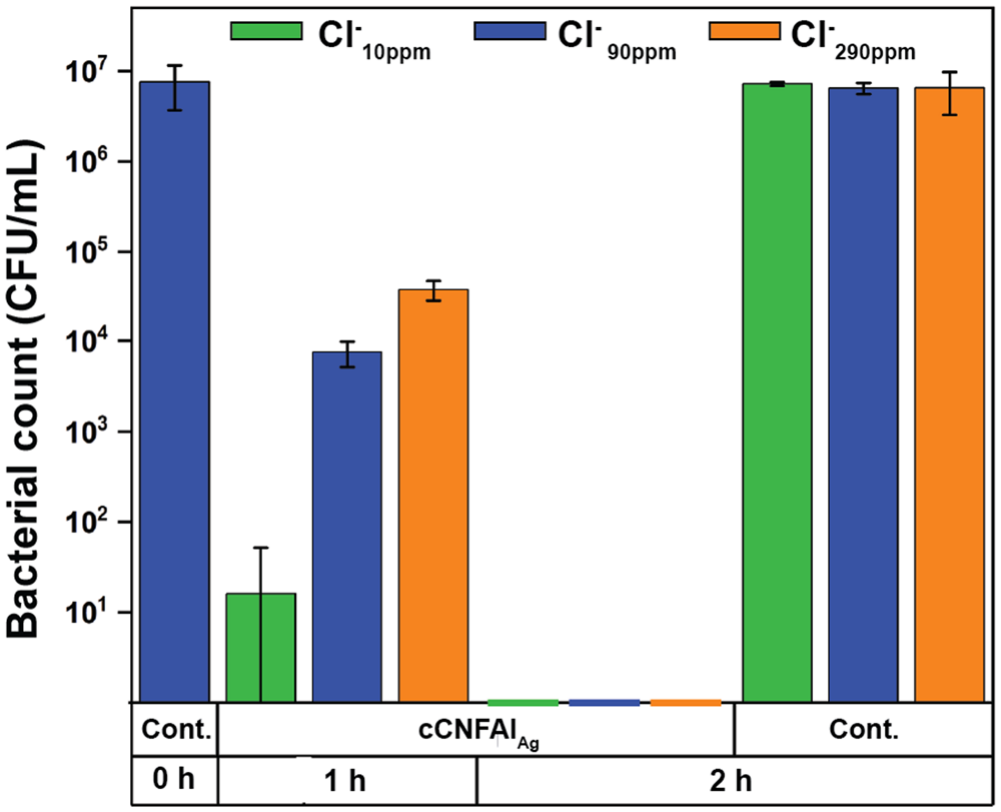
Influence of chloride concentration. The antibacterial activity of cCNFAl_Ag_ composite against *E. coli* in Cl^−^_10ppm_, Cl^−^_90ppm_, and Cl^−^_290ppm_ simulated freshwaters.

Tests were continued with *E. coli* by studying the situation with no culture medium added in Cl^−^_10ppm_, Cl^−^_90ppm_, and Cl^−^_290ppm_ simulated freshwaters (Fig. 5). As a background, antibacterial tests with AgNPs and silver-releasing materials have previously been performed in different bacterial culture media^50,51^, phosphate buffer^3,16^, PBS buffer^18,33^, simple aquatic media (for example containing only NaCl and KCl^52^), dechlorinated tap water^53^ or deionized water^32^. However, in these cases, [Cl^−^] is typically either very high or very low compared to freshwaters. Several studies have also been conducted using natural drinking waters or freshwaters^54–56^. However, [Cl^−^] is often not reported or considered when interpreting results. Since the composition of water strongly affects the antibacterial efficiency, it is difficult to compare the results of these studies, thus urging the present studies.

The results in Fig. 5 indicate that, as expected, the absence of residual culture medium enhanced the antibacterial activity against *E. coli* compared to the situation where nutrients were present. After 1 h of incubation in the absence of nutrients in Cl^−^_90ppm_, the cCNFAl_Ag_ composite caused 2.9 log_10_ reduction in bacterial plate count compared to 0.6 log_10_ reduction in the presence of nutrients. When nutrients were present, it can be assumed that both the bacteria growth and complexation of Ag^+^ with the organic components of LB medium could reduce the antibacterial effect. In natural waters, bacterial growth is typically limited by low concentrations of appropriate carbon and energy sources^57^. Therefore, the conditions without nutrients are assumed to be closer to the state of bacteria populations in natural waters compared to water with added nutrients. The results also indicated good viability of bacteria in the control samples for the duration of the experiments, thus confirming that the antibacterial effects observed were due to the cCNFAl_Ag_ composite.

For achieving an antibacterial effect in water with silver, the release of Ag^+^ is crucial. Water chemistry can impact the kinetics of the release and speciation of Ag^+^^8^. Ag^+^ can form complexes with Cl^−^ leading to the formation of both insoluble and soluble AgCl_x_^(x-1)−^ species depending on the Cl/Ag ratio^58^. Thus, presence of Cl^−^ can reduce the amount of free Ag^+^ and consequently the antibacterial effect can be reduced^59^. Therefore, the influence of [Cl^−^] on the antibacterial activity of cCNFAl_Ag_ against *E. coli* was evaluated by comparing the antibacterial activity of the composite in Cl^−^_10ppm_, Cl^−^_90ppm_, and Cl^−^_290ppm_ simulated freshwaters. The amount of viable bacteria after 1 h contact time increases with increasing [Cl^−^], as observed in Fig. 5. This clearly indicates that decrease of [Cl^−^] increases the antibacterial efficacy of the cCNFAl_Ag_ composite. After 2 h contact time the live bacteria count in all the simulated freshwaters was 0, indicating that with longer contact times the [Cl^−^] is not as crucial for the antibacterial efficiency of cCNFAl_Ag_ as with shorter contact times, thus demonstrating potential for water disinfection.

Several studies have investigated the influence of [Cl^−^] on the antibacterial activity of free AgNPs in the colloidal form^24,25,35,58^. Increased [Cl^−^] was found to shift the dominant aqueous silver species to AgCl_x_^(x-1)−^ which were found to be less toxic to *E. coli*, thus reducing the antibacterial efficiency of AgNPs. As the charge on the dominant species AgCl_x_^(x-1)−^ decreased, the antibacterial efficiency also decreased^35^. Low concentrations of Cl^−^ (12.5 ppm) have been reported to have no effect on the antibacterial properties of AgNPs^24^. The antibacterial effect of polyvinylpyrrolidone coated AgNPs on *E. coli* was significantly decreased in [Cl^−^] of 350 ppm^25^. This concentration is in the same range as the [Cl^−^] of the Cl^−^_290ppm_ simulated freshwater used in this study. Another study showed significant reduction in antibacterial activity against *E. coli* in phosphate buffer with 12 ppb Ag^+^ when [Cl^−^] was 75–150 ppm compared to 0–50 ppm^15^.

Typical contact times used in experiments for testing antimicrobial activity of AgNP containing materials in water are less than 1 h^33^, but longer contact times up to 24 h have also been reported^48,60^. In this study, relatively long contact times, 1 h and 2 h, were chosen to see the influence of [Cl^−^] on the antibacterial effectiveness of the cCNFAl_Ag_ composite. However, it should be noted that the initial bacterial concentrations in this study were high compared to typical reported concentrations in natural waters (for instance 10^2^–10^3^ CFU/mL in rural India^59^ and around 1–2 × 10^2^ total coliforms/mL and 10^2^–10^6^ CFU/mL heterotrophic bacteria in river waters in South Africa^61^). Considering this, it can be argued that shorter contact times could be sufficient in real life applications to achieve satisfactory antibacterial effects. The amount of material used in the experiments also influences the antibacterial effect and in this study we used contact times that were suitable to show the antibacterial effect with the material amount used (4 g/L). It should also be noted that the experiment was conducted within two weeks after the preparation of the material. The antibacterial activity of the material was found to decrease during long-term storage of the composite in ambient conditions, which is likely explained by the oxidation of AgNPs over time leading to passivation of the AgNP surfaces. This was evident based on an experiment conducted after one month of the composite preparation. No viable bacteria were detected in Cl^−^_10ppm_ and Cl^−^_90ppm_ after 2 h contact time, but viable bacteria were observed in Cl^−^_290ppm_.

In addition to [Cl^−^], there are several other factors that can influence the antibacterial effect of AgNPs. In natural waters, binding can occur to dispersed organic matter such as humic acid, which reduces the amount of dissolved silver^7^. Thiols present in organic matter have been predicted to compete with Cl^−^^62^. Sunlight irradiation^23^, other electrolytes, pH, carbonate concentration^59^, and dissolved oxygen have also been shown to affect antibacterial efficiency of silver^63^. In this work, we used simulated freshwaters instead of real water samples to quantify the influence of [Cl^−^] to the antibacterial effect of silver-releasing materials based on AgNPs. To apply silver-releasing materials for real-life water purification applications, many other factors including preservation of the AgNPs during storage and the microflora composition of the water to be purified need to be considered as well. These would be interesting aspects to focus on in future studies on silver-based materials for POU water disinfection.

The cCNFAl composite not containing AgNPs was used as a reference material and was found to slightly decrease the bacterial count in Cl^−^_90ppm_ (0.5 log_10_ reduction after 1 h incubation) (Supplementary Fig. S5). This could have been caused by the adsorption of bacteria on the positively charged composite material (surface charge 0.8 mmol/g at pH 7.4). Another possibility is that cCNFAl has minor antibacterial properties due to the inherent cationic charge of the material, as shown in the case of cCNF matrix^21,22^. The antibacterial effect of positively charged materials is based on their interaction with the negatively charged phospholipids in the cell membranes of bacteria. Even though cCNFAl caused a minor decrease of the bacterial count, the antibacterial activity of cCNFAl_Ag_ was significantly higher.

### Silver release and speciation

**Figure 6:**
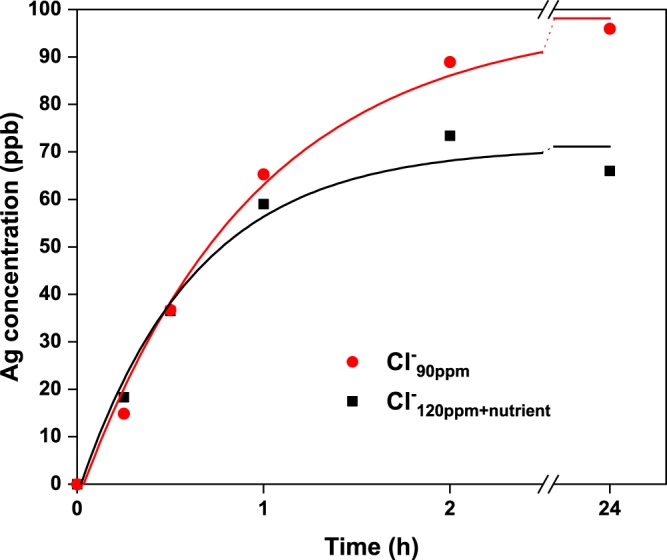
Silver release from cCNFAl_Ag_ composite (4 g/L) in Cl^−^_90ppm_ and Cl^−^_120ppm+nutrient_ simulated freshwaters. Exponential decay curves (increasing form) have been plotted to the data to guide the eye.

The United States Environmental Protection Agency indicates 100 ppb as a health advisory lifetime maximum level of silver in its secondary drinking water regulations^64^. On the other hand, it has been reported that a minimum concentration of silver of 50 ppb is needed to achieve antibacterial effect in typical natural waters^59^. Therefore, a major challenge in silver-based water purification is to develop a material that releases the required amount of Ag^+^ to achieve antibacterial effect but restricts the concentrations to a level regarded safe for human health. Silver dissolution from the cCNFAl_Ag_ composite was determined by inductively coupled plasma mass spectrometry (ICP-MS) in separate tests without bacteria present in Cl^−^_90ppm_ and Cl^−^_120ppm+nutrient_ using the composite concentration of 4 g/L. Figure 6 shows the release of silver from cCNFAl_Ag_ as a function of time, showing that silver dissolution is clearly time dependent and that the silver concentration saturates to below 100 ppb in both Cl^−^_90ppm_ and Cl^−^_120ppm+nutrient_ after 24 h contact time. It can be observed that the amount of dissolved silver species was higher from Cl^−^_90ppm_ than Cl^−^_120ppm+nutrient_. This indicates that components from the culture medium decrease the amount of dissolved silver species from the composite. Free Ag^+^ ion percentages 64.9, 15.3 and 3.9 of total Ag, for Cl^−^_10ppm_, Cl^−^_90ppm_, and Cl^−^_290ppm_ waters, respectively, were calculated based on a computational speciation analysis (Supplementary Table S3). Thus, the amount of free Ag^+^ strongly depends on the composition of the water to be purified, and particularly its Cl^−^ content.

## Discussion

Based on the collected field samples, the [Cl^−^] of potential drinking water sources can vary significantly, thus influencing the antibacterial effect of AgNP embedded materials. In this study, efficient antibacterial activity was achieved when [Cl^−^] was in the low range of concentrations present in possible freshwater sources, e.g. 10 ppm. If the [Cl^−^] was high, the contact time of the material with water needed to be at least 2 h to reach a significant reduction of bacterial load. We see that in order to utilize AgNP embedded materials, it needs to be ensured that the contact time is sufficient or that [Cl^−^] is at a low enough level, which could be achieved by auxiliary methods such as ion exchange or biosorption. In this study, the composite was directly brought into contact with water. For field applications, solutions such as loading the composite into a column or a filter unit of a gravity driven device could be considered. Further studies should also be conducted on the antibacterial capacity of the composite to determine when re-loading of the composite with silver or replacing the material would be necessary.

The results of this study indicate that the conditions of the aqueous environment need to be considered carefully when applying silver-releasing materials to POU water purification. We feel there is a lack of consideration in the case of POU water purification materials based on embedded nanosilver. This often leads to studies of limited significance in terms of real-life applications. We also want to address that mimicking natural aquatic environments in laboratory conditions is challenging, due to which field tests are necessary for actual applications. However, the complexity of natural waters presents several additional challenges. Therefore, conducting laboratory tests with reproducible simulated water compositions, as demonstrated here, is useful when identifying the effects of individual variables, such as [Cl^−^].

## Methods

### Materials and preparation of simulated freshwaters

The cCNF containing quaternary ammonium groups was provided by Betulium Ltd., Finland. The average degree of substitution provided by the manufacturer was 0.1, determined as the amount of substituted glucose units or 0.63 mmol/g of cationic groups. Al_2_(SO_4_)_3_ • 18 H_2_O, was purchased from Sigma-Aldrich (>97%) and Al_2_(SO_4_)_3_ • 14 H_2_O from Aqua Nova Oy (technical grade, >90%). Chemicals used to prepare simulated freshwater (NaCl, MgSO_4_, KNO_3_, NaHCO_3_, and CaCl_2_), AgNO_3_, and NaBH_4_ were purchased from Sigma-Aldrich. The water was prepared by dissolving the chemicals into Milli-Q water (Millipore) to achieve the ion composition typical for freshwaters and the pH was adjusted to 7.2–7.4 with 6 M HCl. The composition of Cl^−^_90ppm_ water was formulated using the natural drinking water composition from Sankar *et al*. as a basis, omitting the silicate and fluoride and with the addition of carbonates^19^. Due to this, the chloride and magnesium concentrations are lower than those used by Sankar *et al*. The addition of 1% culture medium with bacteria inoculation resulted in higher sodium and chloride concentrations in Cl^−^_120ppm+nutrient_ compared to Cl^−^_90ppm_.

### Preparation of composites

For the preparation of cCNF-based composites, a method reported for the preparation of chitosan-based composites with embedded AgNPs^19^ was used as a basis. 1.5 L of 1.5 wt-% dispersion of cCNF was prepared and 1.25 L of 0.5 M Al_2_(SO_4_)_3_ was added dropwise under stirring. The mixture was kept under stirring for 3 h. Further, 2 M NaOH was added dropwise to reach pH 9 and stirring was continued for 1 h. The suspension was filtered to collect the precipitate, which was washed extensively with water. Part of the precipitate was separated to act as a control material having no Ag. To form a composite embedded with AgNPs, a part of the above-described precipitate (dry mass of 86 g) was resuspended in 2 L of water and 1.07 L 5 mM AgNO_3_ was added. The solution was kept under stirring for 1.5 h. Then 1.07 L 10 mM NaBH_4_ was added dropwise at <10 °C. The mixture was stirred for 20 min and then left to stand for 20 min. The suspension was filtered to collect the precipitate. The precipitate was washed extensively with water, collected, and dried to a paste with a solid content of 20–30 wt-%. The paste was extruded through a syringe and dried to form pellets noted as cCNFAl_Ag_. Pellets were also formed with the same method from the above-described precipitate without Ag (noted as cCNFAl).

### Characterization of cationic cellulose nanofibrils and composites

The structure of cCNF was imaged with a FEI Tecnai 12 TEM operating at 120 kV. For sample preparation, 3 µL 0.01 wt-% dispersion of cCNF was drop-casted on a copper grid with an ultrathin carbon support film and the excess solution was blotted with a filter paper after 1 min contact time, followed by drying under ambient conditions. Thereafter, 3 µL of 2% uranyl acetate was drop-casted onto the dried cCNF sample to stain the sample. The excess solution was blotted with a filter paper after 1 min of contact time, followed by drying under ambient conditions. FTIR spectra of freeze-dried cCNF samples were recorded with Nicolet 380 FTIR Spectrometer using an attenuated total reflectance (ATR) sampling accessory. The spectra were recorded in the 500–4000 cm^−1^ range with 0.5 cm^−1^ intervals. Adjacent averaging with 10 point window was applied to the data. The pH-dependence of ζ-potential of cCNF was measured from 0.05 wt-% dispersions of cCNF with Zetasizer Nano-ZS90 (Malvern).

The composites were crushed into powder using a mortar and pestle for the SEM imaging, XRD analysis, EDX analysis, and charge determination. The SEM images were taken with a field emission SEM (Zeiss Sigma VP, Zeiss). The crushed samples were mounted on carbon tape on aluminium stubs and images were taken using an acceleration voltage of 1.6 kV with a working distance of 8 mm. Samples were sputtered with 10 nm gold before imaging. EDX analysis was done with a field emission SEM with EDX (JSM-7500F, JEOL). Before analysis the samples were mounted on carbon stubs with copper tape and sputter-coated with 10 nm layer of iridium. The XRD patterns were measured with a Rigaku SmartLab X-ray diffractometer and recorded in the 2θ range of 10°–100° with a scan step of 0.01°. The charge of the composites containing no silver was determined by conductometric titration. The composites were repeatedly washed with 10% NaCl solution to change the counter ions to Cl^−^. The ion-exchanged composites were washed repeatedly with water, dried, and subjected to conductometric titration with 10 mM AgNO_3_. Brunauer-Emmett-Teller (BET) surface areas were measured with a nitrogen sorption apparatus (Tristar II, Micrometrics). Samples were kept in a 105 °C oven overnight and then outgassed for 1 h at 120 °C under nitrogen flow before measurements.

### Bacteria strains

*E. coli* (DSM 1116, ATCC 9637) and *B. subtilis* (1012M15, details provided elsewhere^65^) were used as Gram-negative and Gram-positive model bacteria, respectively. In all antibacterial tests and related sample preparation, the glassware was autoclaved to avoid bacterial contamination.

### Antibacterial tests

Before tests the simulated freshwaters were sterilized by filtering with 0.2 µm sterile syringe filters. *E. coli* and *B. subtilis* cultures were maintained on LB agar plates at 4 °C. 4 mL of LB medium was activated with the *E. coli* and *B. subtilis* cultures and incubation was carried on overnight at 37 °C and 30 °C, respectively, and 220 rpm. For tests comparing the antibacterial activity on the two bacteria strains, OD_600_ values were adjusted by diluting the inocula with LB to a total volume of 0.5 mL to reach starting bacterial concentration between 10^6^ and 10^7^ CFU (colony forming unit)/mL and then suspended in 49.5 mL of Cl^−^_90ppm_ simulated freshwater.

For tests with *E. coli* in the three different simulated freshwaters, 0.2 mL of the preculture was centrifuged (2 min, 5000 rcf) and the medium was carefully removed and the cells were resuspended in 0.5 mL of Cl^−^_10ppm_, Cl^−^_90ppm_, and Cl^−^_290ppm_ simulated freshwaters. The centrifuging was repeated and the cells were suspended in the simulated freshwaters (resulting in a total volume of 50 mL). Similar pretreatment of inocula was attempted with *B. subtilis* but the viability of the bacteria was found to suffer due to complete removal of the nutrients.

The antibacterial tests were conducted in 250 mL Erlenmeyer flasks using a composite concentration of 4 g/L. Controls without any composite were also conducted for each test. The flasks were shaken at 100 rpm at 37 °C and 30 °C for *E. coli* and *B. subtilis*, respectively. For plating, dilutions were made if needed in simulated freshwater to achieve countable colonies. 50 µL of sample was spread onto LB agar plates and three parallel plates were made for each sampling. The plates were incubated overnight at 37 °C and 30 °C for *E. coli* and *B. subtilis*, respectively, and the colonies were counted. No significant change in pH of the simulated freshwaters was observed after 2 h contact with the cCNFAl_Ag_ composite and *E. coli* (pH change was less than ± 0.1 units).

### Dissolution of silver from composites

The Ag dissolution was determined in separate experiments without bacteria using 4 g/L cCNFAl_Ag_ concentration in Cl^−^_90ppm_ and Cl^−^_120ppm+nutrient_. The flasks were incubated at 37 °C and 100 rpm. Samples were taken at 15, 30, 60, 120 min, and 24 h. Ag concentrations of all samples were analyzed with ICP-MS (PerkinElmer, NexION 300X). The samples were acidified with 5% (vol.) concentrated HNO_3_ (68–70%) before analysis.

The amounts of free Ag^+^ in the simulated freshwaters were calculated using an ion speciation software (PHREEQC). The calculations were made with total Ag concentration of 100 ppb.

## Supplementary information

Supplementary information
