# Supplementary material for: Effects of Chloride Concentration on the Water Disinfection Performance of Silver Containing Nanocellulose-based Composites
Source: Sci Rep. 2019 Dec 20;9:19505. doi: 10.1038/s41598-019-56009-6 (PMC6925197; doi:10.1038/s41598-019-56009-6)
Supplement: Supplementary file 1 — Supplementary information [file 41598_2019_56009_MOESM1_ESM.docx]

**Supporting Information**

**Effects of Chloride Concentration on the Drinking Water Disinfection Performance of Silver Containing Nanocellulose-based Composites**

Janika Lehtonen^1^, Jukka Hassinen*^,2^, Riina Honkanen^3^, Avula Anil Kumar^4^, Heli Viskari^1^, Anu Kettunen^3^, Nikolaos Pahimanolis^5^, Thalappil Pradeep^4^, Orlando J. Rojas^1,2^ and Olli Ikkala*^,1,2^

^1^ Department of Bioproducts and Biosystems, School of Chemical Engineering, Aalto University, P. O. Box 16300, FI-00076 Aalto, Espoo, Finland.

^2^ Department of Applied Physics, School of Science, Aalto University, P. O. Box 16300, FI-00076 Aalto, Espoo, Finland.

^3^ Industrial Water Ltd., Moreenikatu 2 B, FI-04600, Mäntsälä, Finland.

^4^ DST Unit of Nanoscience (DST UNS) and Thematic Unit of Excellence (TUE), Department of Chemistry, Indian Institute of Technology Madras, Chennai 600036, India.

^5^ Betulium Ltd., Tekniikantie 2, FI-02150, Espoo, Finland.

* Corresponding authors

E-mail: [olli.ikkala@aalto.fi](mailto:olli.ikkala@aalto.fi)

E-mail: [jukka.hassinen@aalto.fi](mailto:jukka.hassinen@aalto.fi)

**SUPPORTING INFORMATION CONTENT**

Total number of pages: 12

Total number of figures: 5

Total number of tables: 3

**TABLE OF CONTENTS**

| **Supporting items** | **Title** | **Page no.** |
| --- | --- | --- |
| Table S1 | The ionic compositions of simulated drinking waters used for antibacterial tests | S3 |
| Table S2 | Cl^-^ concentration, *E. coli* count, and total plate count present in the samples collected from IITM campus | S4-S5 |
| Table S3 | Silver speciation in simulated freshwaters used in the study | S6 |
| Figure S1 | ζ-potential of cationic CNF in pH range 3-11 | S7 |
| Figure S2 | X-ray powder diffraction patterns of cCNFAl_Ag_ composites | S8 |
| Figure S3 | SEM-EDX elemental mapping of cCNFAl_Ag_ composite | S9 |
| Figure S4 | Titration curve for cCNFAl composite | S10 |
| Figure S5 | Antibacterial test with cCNFAl | S11 |
| Supporting References | References used for Supporting Information | S12 |

**Supporting Table 1**

# Ionic compositions used for the preparation of simulated freshwaters

**Table S1.** The ionic compositions of simulated freshwaters used for antibacterial tests.

|  | Cl^-^_10ppm_ | Cl^-^_90ppm_ | Cl^-^_290ppm_ | Cl^-^_120ppm+nutrient_^*^ |
| --- | --- | --- | --- | --- |
| Ion | ppm | ppm | ppm | ppm |
| Cl^-^ | 10 | 89 | 290 | 120 |
| SO_4_^2-^ | 33 | 33 | 33 | 33 |
| NO_3_^-^ | 1.8 | 1.8 | 1.8 | 1.8 |
| Carbonates | 54 | 54 | 54 | 54 |
| Na^+^ | 21 | 46 | 180 | 66 |
| Mg^2+^ | 8.3 | 8.3 | 8.3 | 8.3 |
| K^+^ | 1.2 | 1.2 | 1.2 | 1.2 |
| Ca^2+^ | 5.6 | 28 | 28 | 28 |

*Addition of 1% LB medium with bacteria inoculation was taken into account. This composition was used for the test shown in Figure 4.

**Supporting Table 2**

# Sample collection from IITM campus area and the determination of Cl^-^ concentration and bacterial count

Water samples were collected in September 2017 from various sources in the campus area of Indian Institute of Technology Madras (IITM) based in Chennai, India. Samples were collected into sterile plastic bottles and delivered to CVR Labs Pvt. Ltd., Chennai, India for the analyses of Cl^-^ concentration, *Escherichia coli* count and total plate count of bacteria, which are presented in Table S2. The IITM Temple lake sample represents a surface water source. The metro water input sample represents potable inlet water that consists mainly of water brought by trucks to the campus. The water in these trucks originates from different sources and is difficult to track back. The inlet water may also consist of water supplied by the Chennai Metropolitan Water Supply and Sewerage Board (CMWSSB). The metro output sample consists of the same water as metro water input but may also consist of the bore well water. For both open well waters, the main source was rainwater but they also consisted partly of incoming filtrates through the walls of the open wells. The lake water sample and both open well waters represented a typical type of water wherein point-of-use water disinfection could be applied to produce potentially potable water. These waters were found to contain *E. coli*.

**Table S2.** Cl^-^ concentration, *E. coli* count, and total plate count in the samples collected from IITM campus area, Chennai, India. MPN = most probable number.

| Sampling place | Cl^-^ concentration (ppm) | *E. coli* (MPN/100 mL) | Total plate count 22 °C (CFU/mL) |
| --- | --- | --- | --- |
| IITM Temple lake | 92 | 70 | 1500 |
| Open well water (near IITM swimming pool) | 1132 | 110 | 31000 |
| Open well water (near Ocean Engineering Department) | 132 | 280 | 57000 |
| Main gate Metro water input | 125 | 0 | 64 |
| Main gate Metro water output | 254 | 0 | 67000 |
| Bore well (main gate) | 157 | 0 | 100 |
| Output IITM drinking water facility | 19 | 0 | 3500 |

**Supporting Table 3**

# Silver speciation in simulated drinking waters used in the study

The speciation analysis of silver was performed with PHREEQC Interactive, version 3.3.12.12704 (Table S3). The additional ions (mainly Na^+^ and Cl^-^) originating from LB medium were separately considered. It should be noted that LB medium also contains organic components that can effect silver speciation, due to possible complexation between the organic components and silver. However, this was not considered in the speciation analysis. Silver concentration of 100 ppb was used in the speciation analysis. It was observed that the concentration of silver did not affect the speciation in the range between 0-200 ppb.

**Table S3.** Silver speciation in simulated drinking waters used in the study.

|  | **Simulated drinking waters used in the study** | | | |
| --- | --- | --- | --- | --- |
|  | **Cl^-^_120ppm+nutrient_** | **Cl^-^_10ppm_** | **Cl^-^_90ppm_** | **Cl^-^_290ppm_** |
|  | % of total Ag | % of total Ag | | |
| $\mathbf{Ag}^{\boldsymbol{+}}$ | 11.4 | 64.5 | 15.3 | 3.9 |
| $\mathbf{AgCl}$ | 68.2 | 34.3 | 69.2 | 55.1 |
| $\mathbf{AgCl}_{\boldsymbol{2}}^{\boldsymbol{-}}$ | 20.3 | 0.9 | 15.4 | 40.6 |
| $\mathbf{AgSO}_{\boldsymbol{4}}^{\boldsymbol{-}}$ | 0.0 | 0.3 | 0.1 | 0.0 |
| $\mathbf{AgCl}_{\boldsymbol{3}}^{\boldsymbol{2-}}$ | 0.1 | 0.0 | 0.0 | 0.4 |
| **Ag (total)** | 100.0 | 100.0 | 100.0 | 100.0 |

**Supporting Information 1**

**ζ-potential of cationic cellulose nanofibrils**

**Figure S1.** ζ-potential of cationic CNF in pH range 3-11.

**Supporting Information 2**

# Powder X-ray diffraction of cCNFAl_Ag_





**Figure S2.** X-ray powder diffraction patterns of cCNFAl_Ag_ composites prepared from high purity Al_2_(SO_4_)_3_ (red curve) and technical grade, Al_2_(SO_4_)_3_ (black curve). The reflections from the sample prepared from high-purity Al_2_(SO_4_)_3_ originate from nanocrystalline boehmite phase (reflection positions from literature marked by blue lines^1^). The broad reflections from the sample prepared from technical grade Al_2_(SO_4_)_3_ indicated presence of an amorphous form of the material.^2^ The reflections from silver nanoparticles are absent due to the small concentration of silver in the composite (theoretically 0.67 wt%).

**Supporting Information 3**

**SEM-EDX spectrum of cCNFAl_Ag_**


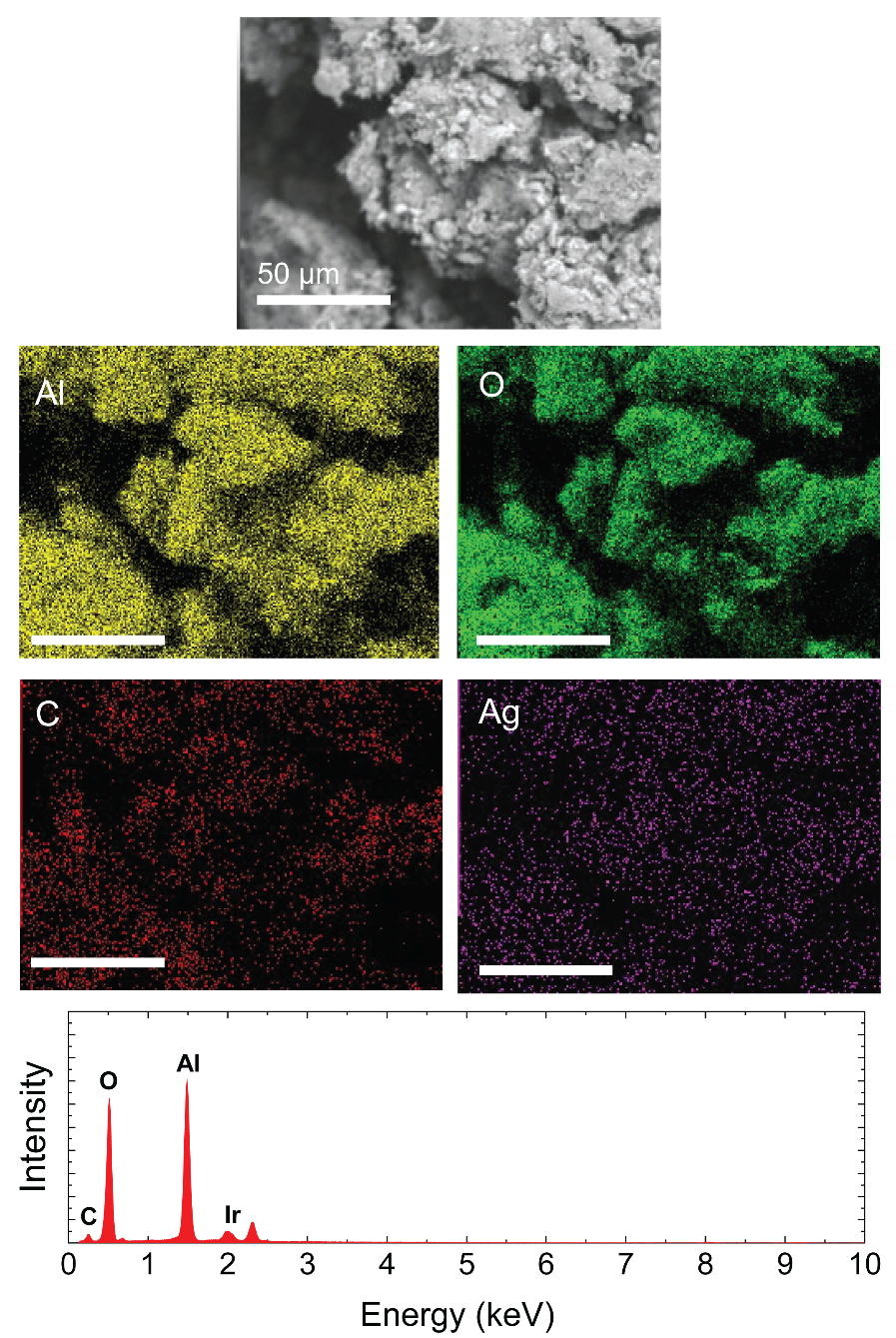


**Figure S3.** SEM-EDX spectrum of cCNFAl_Ag_ composite. Silver could not be detected on the measured EDX spectrum due to its small concentration in the composite (theoretically 0.67 wt%). Iridium was used for coating the sample.

**Supporting Information 4**

# Charge of the cCNFAl composite





**Figure S4.** Titration curve for cCNFAl composite.

**Supporting Information 5**

# Antibacterial test with cCNFAl

To test the antibacterial performance of the composite without AgNPs (cCNFAl), 4 g/L of the composite was incubated in Cl^-^_90ppm_ simulated drinking water and samples were taken at 15 min and 1 h contact times. For inoculation of *E. coli* and plating of the samples, the same procedure was followed as described in the methods section.


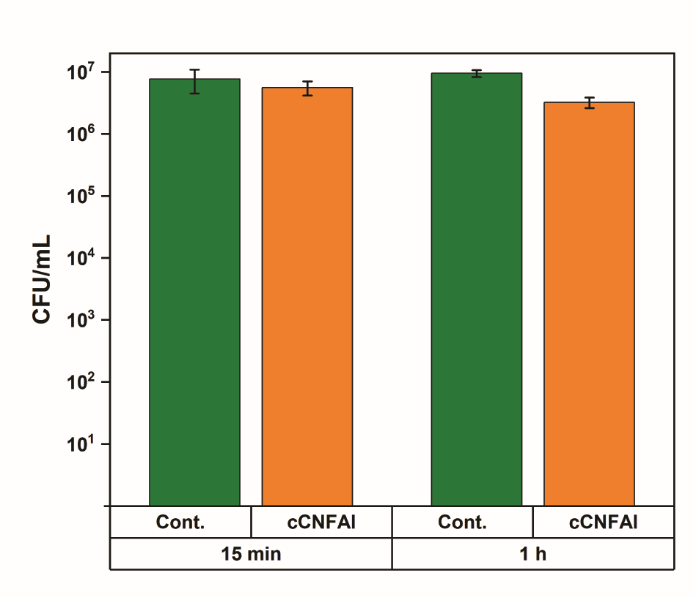


**Figure S5.** Antibacterial activity against *E. coli* of cCNFAl composite (4 g/L) in Cl^-^_90ppm_ simulated drinking water.

**Supporting references**

(1) Hill, R. J. Hydrogen Atoms in Boehmite. a Single Crystal X-Ray Diffraction and Molecular Orbital Study. *CLAYS CLAY MINER. Clays Clay Miner.* **1981**, *29* (6), 435. https://doi.org/10.1346/CCMN.1981.0290604.

(2) Shen, L.; Hu, C.; Sakka, Y.; Huang, Q. Study of Phase Transformation Behaviour of Alumina through Precipitation Method. *J. Phys. D. Appl. Phys.* **2012**, *45* (21). https://doi.org/10.1088/0022-3727/45/21/215302.
